# A new species of *Bolitoglossa* (Amphibia, Caudata) from the Sierra de Juárez, Oaxaca, Mexico

**DOI:** 10.3897/zookeys.185.1146

**Published:** 2012-04-23

**Authors:** Sean M. Rovito, Gabriela Parra-Olea, Dana Lee, David B. Wake

**Affiliations:** 1 Departamento de Zoología, Instituto de Biología, Universidad Nacional Autónoma de Mexico, AP 70-153, Ciudad Universitaria, CP 04510 Mexico DF, Mexico; 2 Museum of Vertebrate Zoology and Department of Integrative Biology, University of California, Berkeley, CA 94720-3160, USA

**Keywords:** Caudata, Mexico, morphology, Plethodontidae, Oaxaca, taxonomy

## Abstract

We describe a new species of *Bolitoglossa* (*Nanotriton*) from the Sierra de Juárez and Sierra Mixe of Oaxaca, Mexico. *Bolitoglossa chinanteca* **sp. n.** is distinguished from the three other species in the subgenus *Nanotriton* by its more robust body, by having substantial numbers of maxillary teeth and differences in relative head width, foot width, and limb length. The new species occurs in sympatry with *Bolitoglossa (Nanotriton) rufescens* at the type locality. The description of another species of salamander from the Sierra de Juárez is noteworthy, given the already high plethodontid salamander species richness of the region.

## Introduction

The genus *Bolitoglossa*, with 117 described species (AmphibiaWeb 2011), is by far the largest genus within the order Caudata. It has the widest range of any tropical salamander genus, from the lowlands of southern Tamaulipas, Mexico to Brazil and Bolivia in South America. The monophyly of *Bolitoglossa* is well supported on molecular (Parra-Olea et al. 2004) as well as morphological grounds. The lack of a sublingual fold, short ceratohyals, partially or fully webbed feet, and fused distal tarsal 4 and 5 characterize all the species of the genus (Wake 1966; Wake and Elias 1983).

Parra-Olea et al. (2004) used mtDNA sequence data analyzed phylogenetically to subdivide *Bolitoglossa* into seven subgenera: *Bolitoglossa*, *Eladinea*, *Magnadigita*, *Mayamandra*, *Nanotriton*, *Oaxakia*, and *Pachymandra*. The subgenus *Nanotriton* comprises species previously included in the *Bolitoglossa rufescens* group: *Bolitoglossa occidentalis* Taylor 1941, *Bolitoglossa rufescens* Cope 1869, and the recently described *Bolitoglossa nympha* Campbell et al. 2010. The species of *Nanotriton* are small, short-tailed salamanders with small pad-like hands and weakly developed feet, all associated with paedomorphosis (Alberch 1983). These species occur in habitats ranging from sea-level lowland forests, to humid cloud forests up to 2000 m in elevation.

In this paper we describe a new species of the subgenus *Nanotriton* from the Sierra de Juárez and Sierra Mixe, Oaxaca, based on morphological differences from described species and DNA sequence differences from sympatric *Bolitoglossa rufescens* and the other two species in the subgenus. The new species is assigned to *Bolitoglossa* (*Nanotriton*) (Parra-Olea et al. 2004) based on its relatively small body size, fully webbed, pad-like feet with little digital individuation, short tail, overall morphological similarity to other species in the subgenus, and phylogenetic placement with mitochondrial DNA sequence data.

## Materials and methods

External morphology was examined in 17 populations of all known species of *Bolitoglossa* (*Nanotriton*) (Table 1). These specimens represent most of the geographic range of the subgenus from the Atlantic coast of Mexico (Veracruz) to western Honduras. We took 14 measurements that reflect size and proportional shape of the salamanders: distance from snout to posterior end of vent (SVL), tail length (TL), snout to gular fold length (SG), head width at angle of jaw (HW), axilla-groin length (AG), forelimb length (FLL), hind limb length (HLL), shoulder width (SW), right foot width (RFW), head depth (HD), interorbital width (IO), internarial width (IN), tip of snout to anterior corner of eye (rostrum length, RL), diameter of eye opening (ED). Measurements were taken to the nearest 0.1 mm using vernier calipers. We also counted the number of costal grooves separating adpressed limbs (limb interval, LI). We counted total numbers of ankylosed vomerine (VT), premaxillary (PMT) and maxillary teeth (MT) under a dissecting microscope.

**Figure 1.**
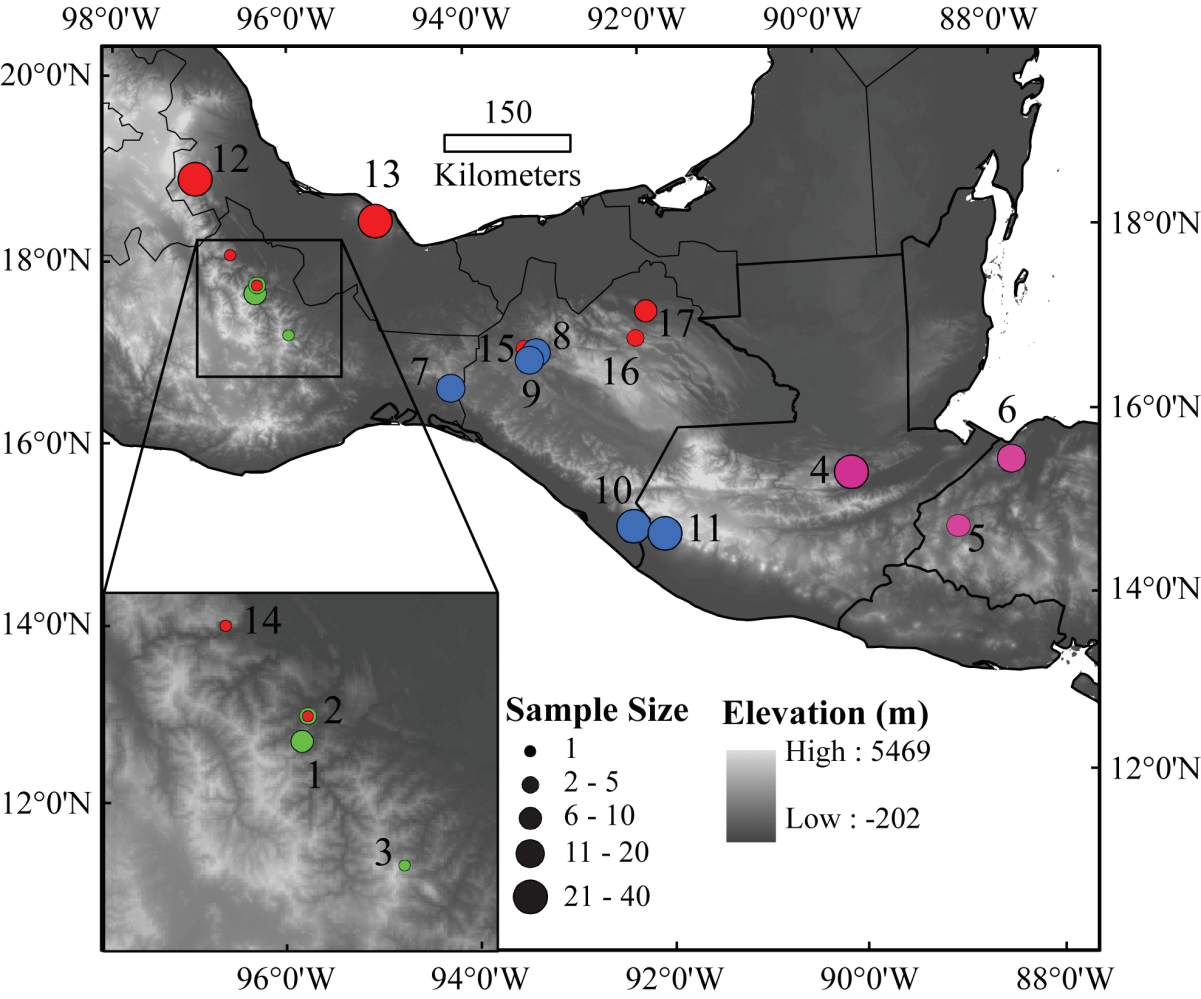
Map of the localities of *Bolitoglossa* (*Nanotriton*) used in the morphological study. Locality numbers correspond to those in Table 1. Green points represent *Bolitoglossa chinanteca* populations, pink points *Bolitoglossa nympha* populations, blue points *Bolitoglossa occidentalis* populations, and red points *Bolitoglossa rufescens* populations.

**Table 1.** Populations and numbers of individuals used in morphological analyses. Numbers of females and males at locality 2 are for *Bolitoglossa chinanteca* and *Bolitoglossa rufescens*, respectively.

Species	Locality	No. females	No. males
*Bolitoglossa chinanteca*	1) Vista Hermosa area, Oaxaca, Mexico	5	3
*Bolitoglossa chinanteca*, *Bolitoglossa rufescens*	2) 10.3 km S of Valle Nacional, Oaxaca, Mexico	2, 1	3, 0
*Bolitoglossa chinanteca*	3) Sierra Mixe, Oaxaca, Mexico	1	0
*Bolitoglossa nympha*	4) Finca El Volcán, Alta Verapaz, Guatemala	5	16
*Bolitoglossa nympha*	5) Santa Rosa de Copán, Copán, Honduras	3	7
*Bolitoglossa nympha*	6) San Pedro Sula area, Cortés, Honduras	6	5
*Bolitoglossa occidentalis*	7) Cerro Baúl, Oaxaca, Mexico	5	6
*Bolitoglossa occidentalis*	8) Copainala area, Tuxtla Gutiérrez, Chiapas, Mexico	10	9
*Bolitoglossa occidentalis*	9) Berriozabal, Chiapas, Mexico	5	6
*Bolitoglossa occidentalis*	10) Tapachula area, Chiapas, Mexico	7	20
*Bolitoglossa occidentalis*	11) Finca Santa Julia, San Marcos, Guatemala	20	20
*Bolitoglossa rufescens*	12) Cuautlapan, Veracruz, Mexico	20	20
*Bolitoglossa rufescens*	13) Catemaco, Veracruz, Mexico	8	19
*Bolitoglossa rufescens*	14) Sierra Mazateca, Oaxaca	1	0
*Bolitoglossa rufescens*	15) Ocozocoautla area, Chiapas, Mexico	2	3
*Bolitoglossa rufescens*	16) Ocosingo, Chiapas, Mexico	1	3
*Bolitoglossa rufescens*	17) Palenque, Chiapas, Mexico	4	6

Species identifications of each population were based on geography and the allozyme results of Larson (1983). *Bolitoglossa nympha*, recently described from the Sierra de Caral on the Guatemala-Honduras border, is currently known only from the type locality (Campbell et al. 2010). The diagnosis for *Bolitoglossa nympha* relies largely on molecular characteristics, which are not currently available for most populations of the *Bolitoglossa rufescens* complex. In Larson’s 1983 allozyme study, populations from Finca El Volcán, Guatemala, the San Pedro Sula area, Honduras and Santa Rosa del Copán, Honduras, which are the geographically closest populations included in our study to the type locality of *Bolitoglossa nympha*, cluster together in a distance-based phylogeny. For this reason, we tentatively treat these three populations as belonging to *Bolitoglossa nympha*. Definitive species identification of populations from eastern Guatemala and Honduras will require detailed molecular study of the complex.

Statistical analyses were run with the program JMP 8 (SAS Institute, Cary, NC, USA). Wilcoxon tests were used to test for differences between group means for selected variables. In order to test for sexual dimorphism within species, variables were tested for normality within each species using the Shapiro-Wilk test. For variables whose distribution did not differ significantly from the normal distribution, a t-test was used to compare the mean for males and females. A Wilcoxon test was used to compare means for males and females of each species for variables whose distributions differed significantly from normal. Significant differences in means between males and females of the same species were found for nearly all variables (see results), indicating that sexual dimorphism exists within these species, so all further analyses were performed separately for males and females.

Multivariate statistical analyses used all variables except LI, MT, and VT, which were not measured in the same units (mm) as the other variables. In order to remove the effect of body size, each variable was regressed against SVL, and the residuals from a linear fit with SVL were used in further analyses. Separate linear fits were used for males and females. Normality of the residuals for each variable was tested using the Shapiro-Wilk test. Given that Discriminant Function Analysis (DFA) can still be used when the assumption of multivariate normality is violated, particularly when the percent of correct classification is high (Klecka 1980), we performed a DFA using residuals from SVL of all variables except TL, which was missing for several individuals of the new species that had missing or regenerated tails.

Because one group (the new species) has a smaller sample size than the total number of variables measured (8 females, 6 males), a Principal Components Analysis (PCA) was used to reduce the dimensionality of the data. The PCA was performed using residuals of the following variables, which passed the normality test: males: SG, FLL, HLL, IO, HW, SW, RFW, ED; females: FLL, HLL, RL, IO, RFW, ED. A second DFA was then performed on the first three principal components.

Although a full molecular analysis of the subgenus *Nanotriton* is beyond the scope of the present work, several mitochondrial sequences were generated in order to compare the new species to other members of its subgenus. We sequenced a specimen (paratype) of the undescribed species (IBH 22535), as well as a specimen of *Bolitoglossa rufescens* (IBH 22536) from the same locality and an individual of *Bolitoglossa occidentalis* (MVZ 194259) for the 16S rRNA (16S, 518 bp) and cytochrome b (cyt b, 809 bp) mitochondrial genes using primers MVZ117 and MVZ98 (Palumbi 1996) for 16S and primers MVZ15 and MVZ16 (Moritz et al. 1992) for cytb. Reactions were run at 94 °C for 2 min, 38 cycles of 94 °C for 30 s, 48 °C for 30 s (16S) or 1 min (cytb), 72 °C for 1 min, with a final cycle at 72 °C for 8 min. We aligned these sequences with available sequences for *Bolitoglossa rufescens* (MVZ 194254) and *Bolitoglossa nympha* (MVZ 194333) from GenBank using the program MUSCLE 3.6 (Edgar 2004) and concatenated alignments for 16S and cytb. We trimmed cytb sequences to a length of 645 bp to match those from GenBank. Individuals of *Bolitoglossa mexicana* (MVZ176838) and *Bolitoglossa hartwegi* (MVZ 263458) were used as outgroups for phylogenetic analysis. We used the program RAxML (Stamatakis 2006) to estimate a phylogeny with maximum likelihood under the GTR+G substitution model in order to determine the relationship of the new Sierra de Juárez species to other members of the subgenus *Nanotriton*. The data were partioned by gene (16S and cytb), and the cytochrome b gene was partitioned by codon position. One thousand bootstrap replicates were performed to assess nodal support. Pairwise distances between species were calculated using PAUP* v4.0 (Swofford 2003). Additionally, we compared the genetic distance between an individual of the new species from the Sierra Mixe (MZFC 16085), for which only a 16S sequence was available in GenBank, to our sample from the Sierra de Juárez.

## Results

Means, standard deviations, and ranges of all measurements and tooth counts are given in Table 2. Our new species showed significant sexual dimorphism for only two variables (HLL, RL), although this result may be partially due to small sample size. Males and females of the other species had significantly different means for the following variables: *Bolitoglossa nympha* – SVL, SG, FLL, HLL, RL, AG, IO, IN, HW, SW, RFW, HD, and VT; *Bolitoglossa occidentalis* – SVL, AG, IO, SW, MT; *Bolitoglossa rufescens* – AG, IO, IN, HW, SW, HD.

**Table 2.** Mean + SD and range for morphological measurements. Number of individuals is indicated when less than the total number for a species.

	***Bolitoglossa chinanteca***		***Bolitoglossa nympha***		***Bolitoglossa occidentalis***		***Bolitoglossa rufescens***	
Measurement	Females *N* = 8	Males *N* = 6	Females *N* = 14	Males *N* = 28	Females *N* = 47	Males *N* = 61	Females *N* = 36	Males *N* = 52
SVL	32.3 + 5.41 (28.4–43.4)	37.6 + 3.19 (33.4–41.2)	37.8 + 3.25 (33.0–43.2)	32.8 + 4.40 (23.0–37.9)	35.9 + 4.86 (26.2–45.6)	33.7 + 2.93 (25.9–38.9)	32.1 + 2.51 (25.9–37.5)	31.1 + 2.34 (25.6–37.2)
TL	28.1 + 5.46 (18.3–34.0) *N* = 6	29.1 + 4.25 (24.0–35.5)	27.5 + 2.74 (23.9–32.3) *N* = 11	25.4 + 4.67 (15.0–33.2) *N* = 25	25.2 + 5.04 (14.7–32.6) *N* = 45	24.8 + 3.00 (19.0–31.9) *N* = 59	22.3 + 2.39 (18.5–27.2) *N* = 35	22.5 + 3.14 (17.7–34.0) *N* =49
SG	9.3 + 1.12 (7.7–10.5)	9.8 + 0.81 (8.8–11.1)	9.2 + 0.71 (8.0–10.3)	8.2 + 0.87 (6.4–9.5)	8.9 + 1.09 (6.6–11.5)	8.58 + 0.67 (7.1–10.2)	8.1 + 0.58 (6.5–9.3)	8.1 + 0.55 (6.6–9.1)
HW	6.1 + 0.79 (5.1-7.1)	6.3 + 0.52 (5.7–6.9)	5.6 + 0.40 (4.7–6.0)	5.0 + 0.58 (3.8–6.2)	5.8 + 0.80 (4.2–7.2)	5.4 + 0.47 (4.2–6.5)	5.1 + 0.34 (4.2–5.9)	5.0 + 0.33 (4.3–5.6)
HD	2.9 + 0.42 (2.5–3.8)	2.9 + 0.53 (2.4–3.9)	2.6 + 0.19 (2.3–3.0)	2.4 + 0.22 (1.9–2.7)	2.6 + 0.30 (1.9–3.4)	2.6 + 0.22 (2.1–3.4)	2.5 + 0.26 (2.1–3)	2.4 + 0.24 (2.0–3.3)
IO	3.4 + 0.44 (2.7–4.1)	3.5 + 0.22 (3.4–3.9) *N* = 5	3.2 + 0.23 (2.9–3.7)	2.9 + 0.39 (2.0–3.5)	3.1 + 0.36 (2.2–4.1)	3.0 + 0.30 (2.3–4.0)	3.0 + 0.28 (2.3–3.5)	2.9 + 0.23 (2.5–3.4)
IN	2.0 + 0.33 (1.5–2.5)	2.3 + 0.13 (2.1–2.5) *N* = 5	1.6 + 0.19 (1.4–2.0)	1.6 + 0.30 (1.0–2.0)	1.59 + 0.33 (1.0–2.3)	1.6 + 0.26 (1.1–2.2)	1.47 + 0.13 (1.2–1.8)	1.4 + 0.23 (1.0–2.0)
ED	2.1 + 0.28 (1.7–2.4)	1.9 + 0.17 (2.2–1.8) *N* = 5	1.8 + 0.15 (1.5–2.0)	1.7 + 0.27 (1.2–2.3)	1.7 + 0.26 (1.2–2.2)	1.7 + 0.24 (1.3–2.2)	1.7 + 0.21 (1.4–2.1)	1.7 + 0.22 (1.4–2.1)
RL	2.6 + 0.32 (2.2–3.1)	2.9 + 0.21 (2.6–3.2) *N* = 5	2.4 + 0.23 (2.0–2.7)	2.2 + 0.32 (1.5–2.7)	2.2 + 0.32 (1.4–3.0)	2.2 + 0.19 (1.9–2.8)	2.1 + 0.17 (1.5–2.3)	2.1 + 0.22 (1.8–3.0)
SW	4.8 + 0.75 (3.4–5.5)	4.4 + 0.28 (4.2–4.9)	4.4 + 0.40 (3.9–5.1)	3.9 + 0.47 (3.0–4.8)	4.2 + 0.54 (3.0–5.5)	3.9 + 0.41 (2.6–4.6)	3.8 + 0.42 (3.0–4.6)	3.5 + 0.30 (2.9–4.2)
AG	18.2 + 3.29 (13.8–23.6	19.3 + 2.03 (16.7–22.0)	20.3 + 3.44 (16.7–29.2)	16.9 + 2.63 (11.6–20.7)	18.7 + 3.01 (12.6–24.5)	16.9 + 1.76 (13.2–19.7)	16.2 + 1.61 (12.4–19.6)	15.4 + 2.28 (11.6–28.1)
FLL	8.4 + 1.04 (6.9–9.8)	8.7 + 0.67 (8.0–9.9)	6.9 + 0.68 (5.7–7.9)	6.3 + 1.02 (3.8–7.5)	7.1 + 1.22 (4.7–9.6)	7.0 + 0.95 (5.3–9.7)	6.4 + 0.63 (5.2–7.9)	6.5 + 0.59 (5.2–8.0)
HLL	8.0 + 0.90 (6.9–9.3)	9.3 + 0.94 (8.6–10.8)	7.2 + 0.74 (5.7–8.2)	6.5 + 1.00 (3.8–7.7)	7.2 + 1.39 (4.7–10.2)	7.0 + 1.07 (4.3–9.5)	6.5 + 0.61 (5.4–7.9)	6.4 + 0.58 (5.1–7.6)
RFW	3.6 + 0.73 (2.6–4.7)	3.9 + 0.45 (3.2–4.4)	3.4 + 0.42 (2.8–4.3)	3.0 + 0.46 (1.7–3.6)	3.3 + 0.61 (2.2–4.4)	3.2 + 0.41 (2.1–4.0)	2.8 + 0.33 (1.8–3.4)	2.8 + 0.36 (2.1–3.6)
LI	1.9 + 0.35 (1–2)	1.4 + 0.80 (0–2)	2.7 + 0.52 (2–4)	2.2 + 0.67 (1–4)	3.3 + 0.58 (2–4)	2.8 + 0.66 (2–5)	2.6 + 0.70 (0.5–4)	2.0 + 0.58 (1–3)
VT	24.6 + 8.10 (11–39)	22.5 + 2.26 (19–25)	19.6 + 3.77 (14–28)	16.3 + 4.50 (10–25)	20.5 + 5.25 (12–34)	17.8 + 5.88 (6–32)	15.3 + 4.76 (5–26)	15.9 + 14.2 (6–33)
PMT	3.3 + 1.37 (2–6) *N* = 6	2.5 + 1.29 (1–4) *N* = 4	0.7 + 0.83 (0–2)	2.4 + 0.92 (1–4)	1.4 + 1.76 (0–9)	1.4 + 1.13 (0–4)	1.8 + 1.00 (0–4)	1.8 + 0.80 (1–4)
MT	23.1 + 10.8 (9–40)	29.8 + 10.68 (21–49)	0 (0)	0 (0)	17.0 + 10.40 (1–42)	11.4 + 9.06 (0–42) *N* = 60	1.4 + 2.91 (0–10) *N* = 35	3.6 + 5.72 (0–20) *N* =51

The Discriminant Function Analysis (DFA) for females using three principal components constructed from residuals from SVL of variables that passed normality tests correctly classified all but one individual of the new species (Table 3). Five of 14 individuals of *Bolitoglossa nympha* were misclassified as *Bolitoglossa occidentalis*, 23 of 46 individuals of *Bolitoglossa occidentalis* were misclassified (12 as *Bolitoglossa nympha* and 11 as *Bolitoglossa rufescens*), and 19 of 36 individuals of *Bolitoglossa rufescens* were misclassified (1 as *Bolitoglossa chinanteca*, 8 as *Bolitoglossa nympha*, 10 as *Bolitoglossa occidentalis*). For males, four of five individuals of the new species were classified correctly while many individuals of the other species were misclassified (Table 3). The number of individuals classified per group differs from the total number of individuals per group because some individuals lack data for measurements such as TL, and the DFA classifies only individuals with data for all included variables. Using residuals from SVL of all variables except TL, misclassification rates were lower (Table 4). For females, all individuals ofthe new species were classified correctly, and only one individual of *Bolitoglossa nympha* was misclassified. For males, all individuals of the new species were classified correctly, while misclassification was higher for the other species (Table 4).

**Table 3.** Discriminant Function Analysis (DFA) results from principal components constructed from residuals of variables that passed normality tests. Rows represent actual species assignments and columns represent predicted group membership from DFA.

**Females**	***Bolitoglossa chinanteca***	***Bolitoglossa nympha***	***Bolitoglossa occidentalis***	***Bolitoglossa rufescens***
*Bolitoglossa chinanteca*	7	0	1	0
*Bolitoglossa nympha*	0	9	5	0
*Bolitoglossa occidentalis*	1	12	23	11
*Bolitoglossa rufescens*	1	8	10	17
Males	*Bolitoglossa chinanteca*	*Bolitoglossa nympha*	*Bolitoglossa occidentalis*	*Bolitoglossa rufescens*
*Bolitoglossa chinanteca*	5	0	0	0
*Bolitoglossa nympha*	1	22	3	2
*Bolitoglossa occidentalis*	0	10	38	11
*Bolitoglossa rufescens*	3	1	13	35

**Table 4.** Discriminant Function Analysis (DFA) results from residuals from SVL for all variables except TL. Rows represent actual species assignments and columns represent predicted group membership from DFA

**Females**	***Bolitoglossa chinanteca***	***Bolitoglossa nympha***	***Bolitoglossa occidentalis***	***Bolitoglossa rufescens***
*Bolitoglossa chinanteca*	8	0	0	0
*Bolitoglossa nympha*	0	13	0	1
*Bolitoglossa occidentalis*	0	4	33	10
*Bolitoglossa rufescens*	0	1	13	22
Males	*Bolitoglossa chinanteca*	*Bolitoglossa nympha*	*Bolitoglossa occidentalis*	*Bolitoglossa rufescens*
*Bolitoglossa chinanteca*	5	0	0	0
*Bolitoglossa nympha*	1	22	3	2
*Bolitoglossa occidentalis*	0	10	38	13
*Bolitoglossa rufescens*	3	1	13	35

**Figure 2.**
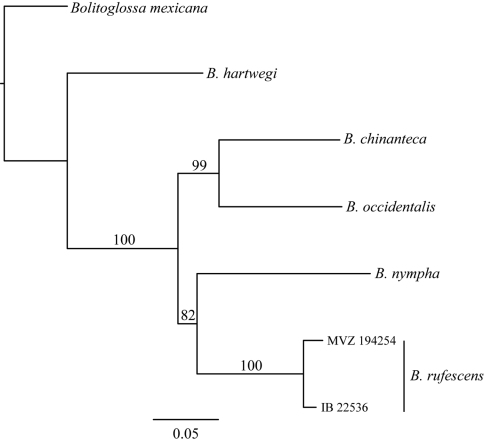
Maximum likelihood gene tree of 16S+cytb genes. Numbers above branches represent support values from 1000 bootstrap replicates.

The maximum likelihood mitochondrial gene tree places *Bolitoglossa chinanteca* as the sister taxon of *Bolitoglossa occidentalis* with strong support (BS=99) (Fig. 2). The GTR distance between individuals of the new species (IBH 22535) and *Bolitoglossa rufescens* from the Sierra de Juárez is 0.08 for 16S and 0.21 for cyt *b*. The two samples of *Bolitoglossa chinanteca* from the type locality and the Sierra Mixe have a GTR distance of only 0.004 for 16S.

Based on the correct classification of nearly all individuals of the new species from the Sierra de Juárez and Sierra Mixe, as well as several differences in external morphology and tooth counts from other species of *Bolitoglossa* (*Nanotriton*) and differences in mtDNA sequence data, these individuals represent an undescribed species. Type specimens are deposited in the Colección Nacional de Anfibios y Reptiles, Instituto de Biología, Universidad Nacional Autónoma de México (IBH).

### Bolitoglossa (Nanotriton) chinanteca sp. n.

urn:lsid:zoobank.org:act:ADAB4FB3-5B4A-4487-B254-EF9E481013B3

http://species-id.net/wiki/Bolitoglossa_chinanteca

Figure 3A–3I

Bolitoglossa occidentalis (in part). Duellman 1960.
Bolitoglossa rufescens (in part). Larson 1983.
Bolitoglossa rufescens . Campbell et al. 2010. Fig. 28A

#### Vernacular name:

Chinanteca Salamander

Salamandra chinanteca

#### Holotype.

Colección Nacional de Anfibios y Reptiles IBH 24708, field number SMR1401, an adult female from 10.3 km south (by rd) of center of Valle Nacional on Hwy. 175, Sierra de Juárez, Oaxaca, Mexico, 676 m elevation, 17.72390°N, 96.32100° W (WGS84 datum), collected by Sean M. Rovito and Dana Lee on 26 October, 2010.

#### Paratypes.

Thirteen specimens, all from Oaxaca, Mexico. 7 females: Sierra de Juárez: IBH 24709, same locality data as holotype; KU 136428, 4.6 km N Vista Hermosa; KU 86616–86617, Villa Hermosa [=Vista Hermosa]; MZFC 5323, Vista Hermosa; MVZ 131152, along Mexico Hwy. 175, vicinity of Vista Hermosa, Distrito Ixtlán; Sierra Mixe: MZFC 16085, Carretera Coconales-Zacatepec; 6 males: Sierra de Juárez: IBH 22535, IBH 24711, IBH 24712, same locality data as holotype. KU 86618, Villa Hermosa [=Vista Hermosa]; UCM 52439, UMMZ 119647, Vista Hermosa.

#### Referred specimens.

None.

#### Diagnosis.

Distinguished from species of all other genera of Neotropical salamanders by the lack of a sublingual fold. Distinguished from species of *Bolitoglossa* (*Magnadigita*) and *Bolitoglossa* (*Oaxakia*) (Parra-Olea et al. 2004) by the presence of fully webbed, pad-like feet and smaller size. Distinguished from species of *Bolitoglossa* (*Pachymandra*) and *Bolitoglossa* (*Bolitoglossa*) by smaller size, smaller hands and feet, and shorter tail, from species of *Bolitoglossa* (*Mayamandra*) by less broad feet, and from *Bolitoglossa* (*Eladinea*) by having a complex tail base in which the transverse processes of the first caudal vertebrae extend forward and cross those of the more anterior vertebra (Parra-Olea et al. 2004). Distinguished from all species of *Bolitoglossa* (*Nanotriton*) by more robust body. Distinguished from *Bolitoglossa nympha* by the presence of maxillary teeth, a relatively wider head (HW/SVL females: *Bolitoglossa chinanteca*: 0.17 + 0.018 vs. *Bolitoglossa nympha* 0.15 + 0.0063, Wilcoxon test, Z = -3.44, p = 0.0006; males: *Bolitoglossa chinanteca*: 0.17 + 0.010 vs. *Bolitoglossa nympha* 0.15 + 0.008, Wilcoxon test, Z = -2.78, p = 0.0055), relatively longer forelimbs (FLL/SVL females: *Bolitoglossa chinanteca*: 0.24 + 0.014 vs *Bolitoglossa nympha*: 0.18 + 0.010, Wilcoxon test, Z = -3.79, p = 0.0002; males: *Bolitoglossa chinanteca*: 0.24 + 0.0067 vs. *Bolitoglossa nympha*: 0.19 + 0.014, Wilcoxon test, Z = -3.77, p = 0.0002), and relatively wider feet (RFW/SVL females: *Bolitoglossa chinanteca*: 0.10 + 0.0081 vs *Bolitoglossa nympha* 0.089 + 0.0077, Wilcoxon test., Z = -2.70, p = 0.0070; males: *Bolitoglossa chinanteca*: 0.10 + 0.0009 vs *Bolitoglossa nympha*: 0.09 + 0.073, Wilcoxon test, Z = -2.87, p = 0.0041). Distinguished from *Bolitoglossa occidentalis* by having more maxillary teeth in males (*Bolitoglossa chinanteca*: 29.8 + 10.7 vs. *Bolitoglossa occidentalis*: 11.4 + 9.1; Wilcoxon test, Z = -3.43, p = 0.0006), a wider head in females (HW/SVL *Bolitoglossa chinanteca*: 0.17 + 0.018 vs. *Bolitoglossa occidentalis* 0.16 + 0.0054, Wilcoxon test, Z = -2.85, p = 0.0043), relatively longer forelimbs (FLL/SVL females: *Bolitoglossa chinanteca*: 0.24 + 0.014 vs *Bolitoglossa occidentalis*: 0.20 + 0.017, Wilcoxon test, Z = -4.40, p < 0.0001; males: *Bolitoglossa chinanteca*: 0.24 + 0.0067 vs. *Bolitoglossa occidentalis*: 0.21 + 0.017, Wilcoxon test, Z = -3.55, p = 0.0004), and relatively wider feet in females (RFW/SVL *Bolitoglossa chinanteca*: 0.10 + 0.0081 vs *Bolitoglossa occidentalis*: 0.092 + 0.0074, Wilcoxon test., Z = -2.14, p = 0.0326). Distinguished from *Bolitoglossa rufescens* by having more maxillary teeth (females: *Bolitoglossa chinanteca*: 23.1 + 10.8 vs. *Bolitoglossa rufescens*: 1.4 + 2.9, Wilcoxon test, Z = 4.19, p < 0.0001; males: *Bolitoglossa chinanteca*: 29.8 + 10.7 vs. *Bolitoglossa rufescens*: 3.6 + 5.7, Wilcoxon test, Z = 6.50, p < 0.0001), relatively longer forelimbs (females: *Bolitoglossa chinanteca*: 0.24 + 0.014 vs *Bolitoglossa rufescens*: 0.20 + 0.015, Wilcoxon test, Z = -4.31, p < 0.0001; males: *Bolitoglossa chinanteca*: 0.24 + 0.0067 vs. *Bolitoglossa rufescens*: 0.21 + 0.015, Wilcoxon test, Z = -3.55, p = 0.0004), relatively wider feet (RFW/SVL females: *Bolitoglossa chinanteca*: 0.10 + 0.0081 vs *Bolitoglossa rufescens*: 0.088 + 0.0079, Wilcoxon test., Z = -3.24, p = 0.0012; males: *Bolitoglossa chinanteca*: 0.10 + 0.0009 vs *Bolitoglossa rufescens*: 0.09 + 0.0074, Wilcoxon test, Z = -3.03, p = 0.0025), and a relatively wider head in females (HW/SVL *Bolitoglossa chinanteca*: 0.17 + 0.018 vs. *Bolitoglossa rufescens* 0.16 + 0.0075, Wilcoxon test, Z = -2.81, p = 0.0049).

#### Description of the holotype.

A large adult female (SVL 37.2). Head broad (HW/SVL 0.15); snout truncate; eyes weakly protuberant, not visible when viewed from below. Maxillary teeth numerous (40 maxillary teeth), 6 premaxillary teeth anterior to line of maxillary teeth, do not pierce lip. Vomerine teeth numerous (29), extending in an irregular row to below the center of the internal nares, forming a more numerous patch near internal nares. Labial protruberances moderately developed. Tail fairly rectangular at base, becoming more rounded only at tip and tapering more sharply on posterior one-third; very weakly constricted at base; relatively short (SVL/TAL 0.78). Limbs relatively short (FLL/SLV 0.24, HLL/SVL 0.22); adpressed limbs separated by approximately 2 costal folds. Hands and feet strongly webbed, with only digit 3 emerging from web. Digits poorly defined except near distal tips; third digit on hands and feet pointed, others rounded; subterminal pad not evident; digits in order of increasing length I-II-IV-III on hands and I-V-II≈IV-III on feet.

**Figure 3.**
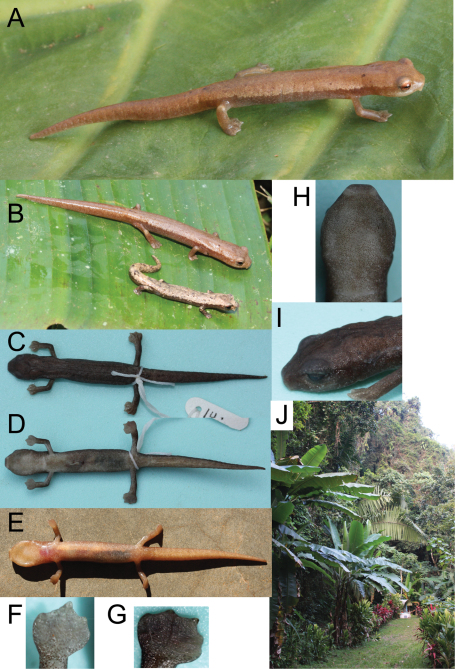
Photographs of the live and preserved holotype. **A** Holotype of *Bolitoglossa chinanteca* **B** Holotype of *Bolitoglossa chinanteca* with a sympatric individual of *Bolitoglossa rufescens* **C** Dorsum and **D** venter of preserved holotype **E** Ventral view of holotype before preservation, showing color in life **F** Right hand, **G** right foot **H** gular region and **I** side view of head of preserved holotype. **J** Photograph of the type locality of *Bolitoglossa chinanteca*, including banana plants where the type series was collected. All photographs by S. M. Rovito.

#### Measurements

**(in mm), limb interval and tooth counts of the holotype.** SVL 37.2; HW 5.6; SG 9.8; HD 2.8; eyelid length 2.5; eyelid width 1.6; eye-nostril distance 2.0; ED 1.8; IO 3.8; IN 2.2; RL 3.0; snout to forelimb 10.6; snout to anterior angle of vent 34.2; AG 19.3; TL 29.2; tail width at base 3.0; tail depth at base 2.8; FLL 8.8; HLL 8.3; width of right hand 2.8; RFW 3.8; length of third toe 0.6; length of fifth toe 0.2; maximum nostril diameter 0.4; SW 5.1. Limb interval 2. Maxillary teeth 40; premaxillary teeth 6; vomerine teeth 29.

#### Coloration (in life)

**of the holotype** (Fig. 3A, 3B, 3E). Dorsum nearly uniform orange-brown with scattered darker brown specks. Dorsal surface of head slightly darker brown between interorbital region and dorsal midline behind eyes, forming a triangle of darker coloration. Rostrum pale brown. Iris coppery. Labial surfaces and sides of head to insertion of forelimb pale brown with tiny cream spots. Dorsal surface of tail similar to dorsum. Dorsal surface of legs, lateral surfaces of body and tail brown with tiny pale flecks scattered throughout. Gular surface pale with cream and brown mottling. Ventral surface pale brown with fine cream mottling. Underside of tail and limbs pale brown with tiny cream and darker brown specks throughout. Underside of feet pale brown.

#### Coloration (in alcohol)

**of the holotype**. Dorsum and dorsal surface of tail dark golden brown with scattered dark brown specks. Head and upper surface of limbs brown. Upper surface of feet golden brown. Sides of body and tail, and head grey-brown with scattered pale flecks. Gular region, underside of forelimbs, and anterior portion of venter (to approximately 2 costal grooves past insertion of forelimbs) cream colored with light brown mottling. Brown mottling more extensive on posterior portion of venter, underside of tail, and underside of hind limbs. Underside of hands pale, underside of feet slightly darker brown.

#### Color variation.

Several of the paratypes exhibit lighter grey dorsal coloration with more dark brown or black specks in alcohol. MZFC 21178 has a lighter reddish brown dorsum, becoming lighter yellow-brown towards the sides of the body, with numerous dark brown flecks throughout. MZFC 21178 has a more yellowish venter, with extensive brown mottling, while IBH 22523 has a darker brown venter with some pale yellow mottling, and a yellowish gular region with brown mottling.

#### Osteology.

A radiograph of a single adult paratype (UMMZ 119647) shows that the species has osteology typical of *Nanotriton*. The hands and feet bear foreshortened digits that taper strongly to their tips. The terminal phalanges are irregular in shape and even number, with a maximal formula of 1-2-3-2 and 1-2-3-2-2. The skull is well formed and has a small dorsal fontanelle between the frontal and parietal bones. The nasal bones are well formed and relatively protuberant from the rest of the skull. Prefrontal bones appear to be present. The preorbital processes of the vomer are long and relatively straight. The vertebral column includes an atlas, 14 trunk, one sacral, two caudosacral and 27 caudal vertebrae. The first caudal vertebra has very elongate transverse processes that arise near the anterior end of the vertebra and extend sharply anterolaterally, strongly overlapping the processes of the last caudosacral vertebra. The long process of the first caudal is bifurcated near its base on one side but less evidently so on the other.

#### Distribution.

This species is known from the Sierra de Juárez, between the small settlement of Vista Hermosa (at approximately 1500 m elevation) and the type locality to the north, along Hwy. 175, as well as from the Sierra Mixe, near the town of Santiago Zacatepec. The two known localities are approximately 70 km (by air) apart. The species presumably occurs on the Atlantic slopes of the Sierra de Juárez and the Sierra Mixe between known populations, and perhaps more widely in the Sierra Mixe.

#### Natural History.

All specimens of *Bolitoglossa chinanteca* for which information is available were collected in the axils of banana plants during the day, and on vegetation at night. The species is presumably arboreal, like other members of the subgenus *Nanotriton*.

#### Etymology.

This species is named after the Chinanteco people from the municipalities of Santiago Comaltepec and San Pedro Yolox (Sierra de Juárez) in the Chinantla region of Oaxaca, where most specimens were collected. The language spoken in Santiago Comaltepec is also called Chinanteco.

## Discussion

The *Bolitoglossa rufescens* group (subgenus *Nanotriton*, following Parra-Olea et al., 2004) long included only two species (*Bolitoglossa rufescens* and *Bolitoglossa occidentalis*) (the taxon *Bolitoglossa bilineata* was synonymized with *Bolitoglossa occidentalis* by Wake and Brame (1969)). Populations assigned to *Bolitoglossa rufescens* and *Bolitoglossa occidentalis* showed high levels of genetic divergence from one another (Larson 1983). The diminutive body size of these animals, coupled with variation in traits considered to be diagnostic for species (such as the presence or absence of maxillary teeth (Larson 1983)), hindered the taxonomic recognition of additional species within the complex. While *Bolitoglossa occidentalis*, *Bolitoglossa rufescens*, and *Bolitoglossa nympha* strongly resemble each other in overall external morphology (as evidenced by high misclassification rates of these species in the DFA), *Bolitoglossa chinanteca* is easily distinguished from these three species by its more robust body, as well as by the combination of characters given above. The occurrence of *Bolitoglossa chinanteca* and *Bolitoglossa rufescens* at the type locality of *Bolitoglossa chinanteca* is the second demonstrated instance of sympatry between two members of the subgenus *Nanotriton*, which further strengthens the case for the recognition of *Bolitoglossa chinanteca* as a distinct species. Poglayen and Smith (1958) reported *Bolitoglossa occidentalis* and *Bolitoglossa rufescens* from 10 km N San Fernando, Chiapas in the Atlantic drainage, and Larson (1983) showed very close geographic proximity between *Bolitoglossa rufescens* and *Bolitoglossa occidentalis* in the vicinity of Berriozabal, Chiapas.

No information is currently available on the population size or status of *Bolitoglossa chinanteca*, although individuals were found at the type locality on two recent visits. Although the distribution of *Bolitoglossa chinanteca* is not known precisely, a polygon drawn between the three known localities has an area of approximately 255 km^2^. This extent of occurrence, coupled with a decline in extent of occurrence due to habitat destruction, would classify *Bolitoglossa chinanteca* as Endangered under IUCN Red List Criterion B1ab(i) (B1. Extent of occurrence < 5000 km^2^, a. known from <5 localities, b(i). continuing decline in extent of occurrence). The fact that *Bolitoglossa chinanteca* has been taken in banana trees in disturbed habitat, however, suggests that it may tolerate disturbance reasonably well. At this time, it does not appear that *Bolitoglossa chinanteca* qualifies for any of the threatened IUCN categories (CR, EN, VU). This assessment could change if evidence arises that it cannot live away from forest (the banana trees at the type locality are on the forest edge) or that habitat destruction in the region is adversely affecting the species. Because of this, we believe that *Bolitoglossa chinanteca* should be classified as Near Threatened (NT).

The Sierra de Juárez is among the areas of highest species richness for Neotropical salamanders, and morphologically distinct species continue to be described from the region (Hanken and Wake 2001; Parra-Olea et al. 2005) despite decades of taxonomic study of its salamanders (Wake et al. 1992). Not including the nearby Sierra Aloapaneca, the Sierra de Juárez was previously known to contain 18 salamander species of five genera (*Bolitoglossa*, *Chiropterotriton*, *Cryptotriton*, *Pseudoeurycea*, and *Thorius*) (Hanken and Wake 2001; Parra-Olea et al. 2005; Wake et al. 1992); the description of *Bolitoglossa chinanteca* brings the total number to 19. Such a high diversity of salamanders is notable even for Mexico, and highlights the need for continued taxonomic study of the salamanders of the region and of southern Mexico in general.

## Supplementary Material

XML Treatment for Bolitoglossa (Nanotriton) chinanteca

## Appendix 1

Additional specimens examined

*Bolitoglossa occidentalis*: Guatemala: San Marcos: MVZ 100849, 108794, 108804, 108808, 108814, 108818, 115755, 138521, 138536, 138542, 138552, 138553, 138556, 138557, 138566, 138572, 138573, 138577, 138581, 138583, 138584, 138588, 138592, Finca Santa Julia, ca. 1.25 km E, 0.75 km S (by air) San Rafael Pie de La Cuesta; MVZ 130110, 130118, 132039, 132075, 132102, 132106, 102109, 132110, 132114, 132117, 132118, 132121, 132123, 165298, 165305, Finca Santa Julia, ca. 1.5 km SE (by air) San Rafael Pie de La Cuesta; MVZ 160865, Finca Santa Julia, ca. 2 km S San Rafael Pie de La Cuesta. Mexico: Chiapas: MVZ 132442, 132443, 132447–132449, 132451, 132455, 132456, 132458, 132459, 132464–132465, 146461, Cafetal along road from Tapachula de Córdova y Ordóñez to Nueva Alemania, 13.6 km N (by road) junction Mexico Hwy. 200; MVZ 132468, 132470, 132473, 132475–132477, 132483, 132485, 132486, 132490, 132490, Finca Cruz Blanca, along road from Tapachula de Córdova y Ordóñez to Nueva Alemania, 8.0 km N (by road) junction Mexico Hwy. 200; MVZ 161019, 13 km N Tapachula de Córdova y Ordóñez on road to Nuevo Alemania; MVZ 176843, 176905, 23.3 km NNW (by road) Tapachula de Córdova y Ordóñez at Rancho El Naranjo; MVZ 160964, 160965, 7.5 mi N Berriozabal; MVZ 194204, 194205, 194207–194211, 194214, –194216, 194218, 194219, banana grove on side of road from Tuxtla Gutiérrez to Chicoasen, 11.2 km N (by road) San Fernando; MVZ 194222–194224, 194230, 194233, 15.3 km ENE (by road) Copainala, along road to Coapilla; MVZ 194225, 194235–194239, 194243–194245, Cafetal 11.4 km NW (by road) Berriozabal; MVZ 163817, 176857, SE face Cerro Baúl; MVZ 163818, 163819, 16.8 km NW (by road) Rizo de Oro, slopes of Cerro Baúl; MVZ 196013–163015, ridge SE Cerro Baúl, 21 km W Rizo de Oro (in Chiapas); MVZ 194255–194258, 194269, Cerro Baúl, 23 km N (by road) Rizo de Oro.

*Bolitoglossa nympha*: Guatemala: Alta Verapaz: MVZ 161047–161056, 161091–161101; Finca El Volcán, 25 km NW (by road) Senahu. Honduras: Cortés: 115293–115303, Sierra del Espíritu Santo, 10 km W (by air) San Pedro Sula. Copán: MVZ 167983–167988, 16851, 168511, 171068, 171069, 171071, 2 km N Santa Rosa de Copán.

*Bolitoglossa rufescens*: Mexico: Chiapas: MVZ 176882–176885, 30.7 km NNW (by road) Ocosingo; MVZ 176890–176899, 23 km SSW (by road) Palenque, Ruiz Cortines; MVZ 176900–176904 26 km N Ocozocoautla de Espinosa; MVZ 194254, Cafetal 12.4 km (by road) N Berriozabal on road to Cairo. Oaxaca: IBH 24710, 10.3 km S (by rd) of center of Valle Nacional on Hwy. 175; MZFC 15756, Tuxtepec; Veracruz: MVZ 85501–85503, 85505, 85510–85514, 85522–85524, 85526, 85528, 85529, 85531, 85532, 85534, 85537, 85538, 85541, 85546, 85547, 85549, 85552, 85553, in banana and coffee fields W of Cuautlapan on side of hill; MVZ 85543, 85560, 85564, 85565, 85568, 86670, 86675, 85576, 85579, 85581–85583, near Cuautlapan; MVZ 85573, 85574, 163801–163803, 163805–163814; Lake Catemaco, 2.5 km SE Coyame; MVZ 171840, 171841, Ojoxapam, 2.5 km SE Coyame, edge of L. Catemaco; MVZ 163827–163832, 171842, 9.3 km N Catemaco; MVZ 171836, Dos Amates, 12 km N Catemaco; MVZ 185992, Rancho El Encanto, 13 km N Catemaco; MVZ 172684, 172685, Forest above Playa Escondida, 30 km NNE Catemaco; MVZ 222515, Playa Escondida, 18.5 mi N (by road) Catemaco.

## References

[B1] Alberch P (1983) Morphological variation in the neotropical salamander genus *Bolitoglossa*. Evolution 37: 906-919. doi: 10.2307/2408406 28563544

[B2] AmphibiaWeb (2011) Information on Amphibian Biology and Conservation. Available at www.AmphibiaWeb.org [accessed 22.VI.2011]

[B3] Campbell JA Smith EN Streicher J Acevedo ME Brodie ED Jr (2010) New salamanders (Caudata: Plethodontidae) from Guatemala with miscellaneous notes on known species. Miscellaneous Publications of the Museum of Zoology, University of Michigan 200: 1-60.

[B4] Duellman WE (1960) A distributional study of the amphibians of the Isthmus of Tehuantepec, México. University of Kansas Publications, Museum of Natural History 13: 19-72.

[B5] Edgar RC (2004) MUSCLE: a multiple sequence alignment method with reduced time and space complexity. BMC Bioinformatics 5: 1-19. doi: 10.1186/1471-2105-5-113 15318951 PMC517706

[B6] Hanken J Wake DB (2001) A seventh species of minute salamander (*Thorius*: Plethodontidae) from the Sierra de Juárez, Oaxaca, México. Herpetologica 57: 515-523.

[B7] Klecka WR (1980)*Discriminant Analysis* Sage Publications, Beverly Hills, CA, 70 pp.

[B8] Larson A (1983) A molecular phylogenetic perspective on the origins of a lowland tropical salamander fauna 1. Phylogenetic inferences from protein comparisons. Herpetologica 39: 85-99. 10.1111/j.1558-5646.1983.tb00228.x 28556019

[B9] Moritz C Schneider CJ Wake DB (1992) Evolutionary relationships within the *Ensatina eschscholtzii* complex confirm the ring species interpretation. Systematic Biology 41: 273-291.

[B10] Palumbi SR (1996) Nucleic acids II: the polymerase chain reaction. In: Hillis DM Moritz C Mable BK (Eds). Molecular Systematics. Sinauer Associates, Sunderland, MA: 204-247.

[B11] Parra-Olea G García-París M Hanken J Wake DB (2005) Two new species of *Pseudoeurycea* (Caudata : Plethodontidae) from the mountains of northern Oaxaca, Mexico. Copeia 2005: 461-469. doi: 10.1643/CH-04-112R

[B12] Parra-Olea G García-París M Wake DB (2004) Molecular diversification of salamanders of the tropical American genus *Bolitoglossa* (Caudata: Plethodontidae) and its evolutionary and biogeographical implications. Biological Journal of the Linnean Society 81: 325-346. doi: 10.1111/j.1095-8312.2003.00303.x

[B13] Poglayen I Smith HM (1958) Noteworthy herptiles from Mexico. Herpetologica 14: 11-15.

[B14] Stamatakis A (2006) RAxML-VI-HPC: Maximum likelihood-based phylogenetic analyses with thousands of taxa and mixed models. Bioinformatics 22: 2688-2690. doi: 10.1093/bioinformatics/btl446 16928733

[B15] Swofford DL (2003) PAUP*. Phylogenetic Analysis Using Parsimony (*and Other Methods). Sinauer Associates, Sunderland, MA.

[B16] Wake DB (1966) Comparative osteology and evolution of the lungiess salamanders, family Plethodontidae. Memoir of the Southern California Academy of Sciences 4: 1-111.

[B17] Wake DB Brame AH (1969) Systematics and evolution of neotropical salamanders of the *Bolitoglossa helmrichi* group. Contributions in Science (Los Angeles) 175: 1-40.

[B18] Wake DB Elias P (1983) New genera and a new species of Central American salamanders, with a review of the tropical genera (Amphibia, Caudata, Plethodontidae). Contributions in Science (Los Angeles) 175: 1-19.

[B19] Wake DB Papenfuss TJ Lynch JF (1992) Distribution of salamanders along elevational transects in Mexico and Guatemala. Tulane Studies in Zoology and Botany Supplementary Publication 1: 303-319.

